# Editorial

**Published:** 1890-10

**Authors:** 


### Editorial.

### A FEW RANDOM THOUGHTS.

Experience has convinced the writer that much harm results from too many visitors to the sick chamber. Many people are indiscreet and will talk too much in the hearing of the patient, and discuss matters likely to cause excitement, when everything should be calm and quiet. We have thought for a long time that some reform was necessary, and have concluded to take the initiative in undertaking to correct the prevailing custom. Our opinion is that no visitor should be allowed unless especially invited by the family. Outside relatives and friends should alike be excluded in all serious cases. There are many ways through which sympathy and interest can be expressed without any danger to the sick. Kind friends can leave cards in a box at the gate or door, or at the postoffice in the towns and cities. In very important cases the bulletin board should be used, stating the condition of the patient. This, we think, would satisfy all sensible people. Sunday is a bad day for the sick. Everybody dresses up on Sunday, and can conveniently step over to see the sick neighbor. We know that this is a kindness in a great many; we know also that some are prompted by curiosity. We are sure that no one would purposely injure the chances of any one for recovery. Still, good friends, prompted by the highest and best of motives, frequently do great harm. Physicians are occasionally to blame in this matter. Some of them are afraid of incurring the displeasure of the people, and suffer their patients to be annoyed and excited by the presence of crowds in the room. The doctors of the olden time were braver and more independent than those of the present day. Whilst there have been many improvements in most things, we have lost ground in the management of the sick room, in the control of the patient, the family and the people. The old-time doctor was a leader in the community, honored and respected by all. He did not pander to the morbid curiosity of anybody, and never felt it necessary to make long and particular explanations about his patients, their diseases and their chances. His word was the law and was occasionally delivered in rather emphatic language.

The next thought is in reference to nurses. We greatly need skilled or trained nurses. A trained nurse is a luxury. He or she does not talk the patient to death, does not know more than the doctor, does not fret the sick by fidgetting around tucking the clothes and fixing the pillows, does not tell how many people were sick the same way and died.

Now, the greatest improvement that could be made would be to establish a training school in the city of Atlanta, or some other great Southern city, for the education and preparation of skilled nurses. Atlanta, always in the lead, should look to the establishment of such an institution. It would not only be an honor, but one of the most important and useful enterprises ever fostered by the gallant city.

We have one more word to speak in behalf of the sick. There ought to be a chair of dietetics in every medical college. The doctor is never so much at a loss as when he is asked what the patient should eat. He knows but little of the culinary art, and is nearly altogether dependent upon the common cook, who knows nothing of chemistry and physiology, and therefore nothing about the necessary elements for the sustenance of the human body. Doctors usually prescribe extract of beef, gruel, chicken soup, eggnog and milk punch, and they are done. Ladies want to give geletin, blanc mange, tea, &c. We need somebody who knows more than this. Let us have the chair of dietetics.

---

Dr. G. Frank Lydston announces that he will send a copy of his lecture on "Sexual Perversion," to any physician sending him the necessary stamp.

---

The Southern Surgical and Gynæcological Association will meet in Atlanta, Ga., November 11th, 12th and 13th. We hope to see all members here.

---

The Mississippi Valley Medical Association meet in Louisville, Ky., on October 8th, 9th and 10th. Many of the bright lights of our profession will be there and read papers.

The second meeting of the Tri-State Medical Association will be held in Chattanooga, Tenn., October 14th, 15th and 16th. Papers are expected from many of our ablest men. The Association have arranged with the railroads for a one-and-a-third fare, for the round trip.

### A $200,000 LIBEL SUIT.

Suit has been entered by William Radam, manufacturer of Radam's Microbe Killer, against the *Druggists Circular*, of New York, for $200,000 damages, the largest amount so far as heard from that was ever asked for in a libel suit of this kind.

The pleadings show that the action is brought to recover damages claimed to have been done the business of the plaintiff by an article published in the *Druggists Circular* for September, 1889. This article gave the result of an analysis of the Microbe Killer, made by Dr. R. G. Eccles, a prominent chemist of Brooklyn, who stated that an identical preparation could be made by the following formula:

| | |
|---|---|
| Oil of vitriol, (impure) - - - | 4 drams. |
| Muriatic acid, (impure) - - - | 1 dram. |
| Red wine, about - - - | 1 ounce. |
| Well or spring water, - - - | 1 gallon. |

This mixture, it was alleged, could be made at a cost of less than five cents per gallon for which Radam charged three dollars.

It was further alleged that while when properly used sulphuric acid, the principal constituent of the Microbe Killer, was a valuable medicine, it was, when taken without due caution or advice, a slow but certain comulative poison; and the theories advanced by Radam, as to the causes of diseases and the proper method of treatment, were alleged to be totally erroneous. Col. Robert G. Ingersoll, the famous lecturer, is the counsel for the plaintiff.

The *Druggists Circular*, which is published at 72 William street, New York, expresses a desire to hear of any case in which unfavorable results have followed the administration of the Microbe Killer or of any other fact that would be interesting under the circumstances. They claim to have published this analysis without malice and with the sole intention of protecting the public from the loss of their health and money by the use of a dangerous nostrum.—*Druggists Circular.*

